# Supplementary material for: Genome-wide CRISPR Screens in T Helper Cells Reveal Pervasive Crosstalk between Activation and Differentiation
Source: Cell. 2019 Feb 7;176(4):882–896.e18. doi: 10.1016/j.cell.2018.11.044 (PMC6370901; doi:10.1016/j.cell.2018.11.044)
Supplement: Data S2. Processed Data from All the Steps of the Analysis, Related to Figure 1 [file mmc2.zip › supplemental data/motif analysis/Pparg_homer/homerResults/motif6.similar.html]

motif6

## Information for motif6

A
C
T
G
G
A
T
C
G
A
C
T
A
C
T
G
A
G
C
T
C
T
A
G
A
C
T
G
A
G
C
T
C
G
A
T
C
G
A
T
  
Reverse Opposite:  

G
C
T
A
G
C
T
A
T
C
G
A
A
G
T
C
A
G
T
C
T
C
G
A
A
G
T
C
C
T
G
A
C
T
A
G
T
G
A
C
  

|  |  |
| --- | --- |
| p-value: | 1e-247 |
| log p-value: | -5.696e+02 |
| Information Content per bp: | 1.641 |
| Number of Target Sequences with motif | 1833.0 |
| Percentage of Target Sequences with motif | 14.64% |
| Number of Background Sequences with motif | 2225.2 |
| Percentage of Background Sequences with motif | 6.21% |
| Average Position of motif in Targets | 100.6 +/- 52.5bp |
| Average Position of motif in Background | 101.6 +/- 64.4bp |
| Strand Bias (log2 ratio + to - strand density) | -0.1 |
| Multiplicity (# of sites on avg that occur together) | 1.09 |
| Motif File: | file (matrix) reverse opposite |

### Similar de novo motifs found

|  |  |  |  |  |  |  |  |
| --- | --- | --- | --- | --- | --- | --- | --- |
| Rank | Match Score | Redundant Motif | P-value | log P-value | % of Targets | % of Background | Motif file |
| 1 | 0.975 | G C T A T C G A T C G A A G T C A G T C G T C A G T A C G C T A C T A G T G C A T G A C C G T A | 1e-208 | -480.701410 | 13.27% | 5.81% | motif file (matrix) |
| 2 | 0.959 | A G C T A C T G A G C T A C T G A C T G A G C T A G C T C G A T | 1e-202 | -466.898432 | 11.81% | 4.93% | motif file (matrix) |
| 3 | 0.921 | A G C T A G C T A C G T C T A G G A C T A T C G C T A G A G C T | 1e-159 | -367.042095 | 14.85% | 7.69% | motif file (matrix) |
| 4 | 0.712 | A T C G A G T C C T A G T A C G G C A T C G A T C G A T A T G C | 1e-128 | -296.550285 | 35.59% | 25.80% | motif file (matrix) |
| 5 | 0.830 | G C A T G C A T C A T G A G C T C T A G T A C G A G C T T C A G C T G A T G A C | 1e-95 | -219.557681 | 8.50% | 4.26% | motif file (matrix) |
| 6 | 0.695 | G A T C G A C T T G A C T G C A G A T C A G T C G T C A A G T C G T C A G T C A T C A G G T A C | 1e-30 | -70.182413 | 0.38% | 0.04% | motif file (matrix) |
